# Neoplasm seeding in biopsy tract of the musculoskeletal system. A systematic review

**DOI:** 10.1590/1413-78522014220200422

**Published:** 2014

**Authors:** Marcelo Parente Oliveira, Pablo Moura de Andrade Lima, Hilton Justino da Silva, Roberto José Vieira de Mello

**Affiliations:** 1 Universidade Federal do Cariri, Juazeiro do Norte, CE, Brazil, Universidade Federal do Cariri, Juazeiro do Norte, CE, Brazil; 2 Universidade Federal de Pernambuco, Recife, PE, Brazil, Universidade Federal de Pernambuco, Recife, PE, Brazil

**Keywords:** Neoplasm seeding, Biopsy, Sarcoma, Bone neoplasms, Recurrence, Musculoskeletal system

## Abstract

To identify, through a systematic literature review, the characteristics of neoplasm seeding in biopsy performed on the musculoskeletal system. We performed a search on PubMed, MEDLINE, LILACS and SciELO from August to October 2010. We included articles that addressed the neoplasm seeding in biopsy performed on the musculoskeletal system. The search was limited to English, Spanish and Portuguese as publication languages, but it was not limited by year of publication. We retrieved 2858 articles, but only seven were selected based on inclusion and exclusion criteria. Other four papers were found in the references of selected articles, totalizing 11 articles that were used to perform this systematic review. Issues may be raised in the literature: age and gender don't seem to influence the occurrence of neoplasm seeding; without resection of the biopsy tract, the possibility of local recurrence is very real; the influence of the type of tumor in the occurrence of neoplasm seeding is uncertain; it is impossible to conclude whether the closed biopsy technique has a lower chance of neoplasm seeding; it is likely that adjuvant chemotherapy has a protective effect against neoplasm seeding; an unfavorable prognosis is expected according to neoplasm seeding results.

## INTRODUCTION

The approach to tumors of the musculoskeletal system requires the integration of clinical, laboratory, radiographic and histological aspects for accurate diagnosis and management leading to successful treatment. In this regard, the biopsy is pointed out as a fundamental step, being essential for the definitive diagnosis and to identify the histological pattern of tumor.^1^ ^-^ ^3^ Biopsy must offer adequate and representative tissue samples for accurate diagnosis, without however manipulate excessively the lesion in order to avoid modifying the tumor relationship between anatomical compartments and contamination of surrounding tissues with tumor cells.^2^

Most authors experienced in the treatment of musculoskeletal tumors advocate removal of the biopsy tract at the time of surgical resection of the tumor, arguing that this path is potentially contaminated by tumor cells.^1^ ^,^ ^4^ ^-^ ^15^ The resection practice along biopsy proves to be much more grounded in an empirical sense than backed up by scientific studies. Still, vague questions are raised in various studies, untested hypotheses emerging. Among them, that the attempt to obtain multiple samples of tissue at biopsy would be associated with increased dissemination and consequently higher probability of contamination of the biopsy tract.^7^ Another empirically widespread issue is that the percutaneous biopsy technique, by involving less manipulation of the tumor tissue, also implies a lower contamination of the biopsy tract.^4^ ^,^ ^7^ ^,^ ^16^ ^-^ ^18^ It has also been observed that the contamination of the biopsy path is more frequent in soft tissue sarcomas than in cartilaginous and osseous lesions.^13^ it is also believed that neoadjuvant chemotherapy has a protective effect in the control of tumor infiltration in the biopsy site,^17^ ^,^ ^19^ and that this contamination has a negative value in the prognosis of affected patients.^20^

The aim of this systematic literature review is to identify the characteristics of tumor contamination in biopsy path of the musculoskeletal system.

## METHODS

A literature search was performed in PubMed, MEDLINE (1966-1996), MEDLINE (1997-2010), LILACS (Latin American and Caribbean Literature on Health Sciences) and SciELO (Scientific Electronic Library Online) databases from August to October 2010. The search was performed using the intersection of keywords found in DeCS (Descriptors in Health Sciences) and MeSH (Medical Subject Headings): neoplasm seeding and biopsy with their counterparts in English and Spanish at all bases. In addition to these descriptors, we carried out a search with the following intersections of free terms, used because of their relevance to the topic studied: biopsy tract AND musculoskeletal tumors; biopsy tract AND musculoskeletal cancer; and biopsy tract AND musculoskeletal neoplasm, with their corresponding terms in English and Spanish on all databases. Were also consulted the references of selected articles for the search of relevant articles. All articles that addressed tumor contamination in the biopsy tract in musculoskeletal system were also included. Articles that addressed tumoral contamination tumor in tract biopsy performed on systems other than the musculoskeletal, and articles that addressed contamination occurred in tumor sites other than the biopsy tract were excluded. Limits were used for articles in English, Spanish and Portuguese languages. No limits on publication date were used.

## RESULTS

**Figure 1:**
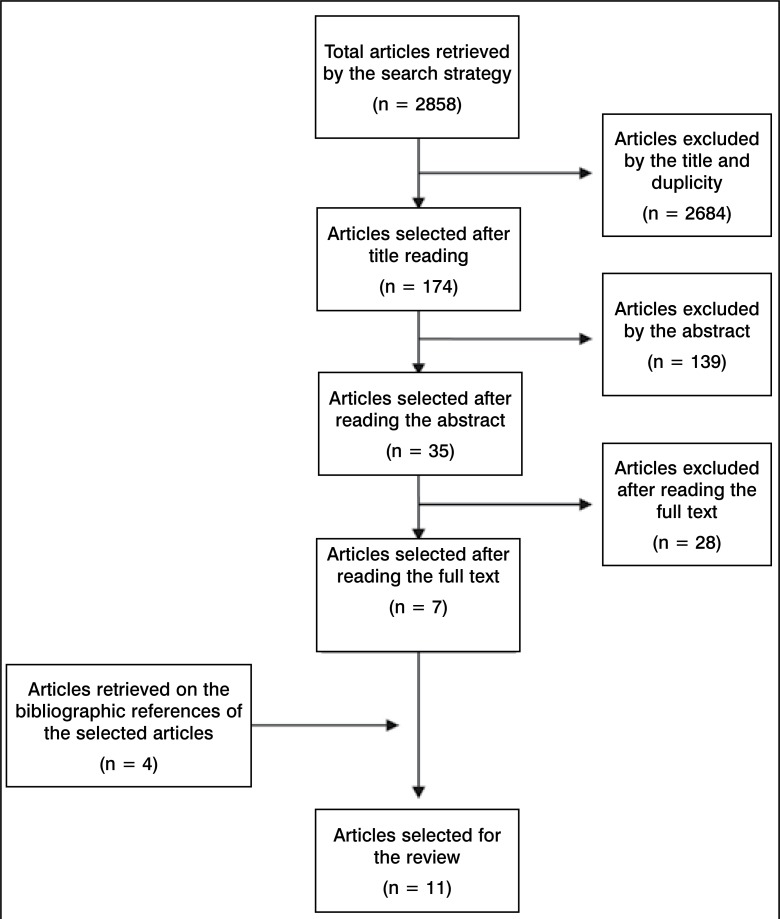
Flow chart of the search strategy used in the selection of articles for the systematic review.

A total of 2,858 articles were retrieved, of which 2,684 were excluded by their title, since they were not adequate to the subject under study or by being duplicated in the databases, leaving 174 papers selected for summary reading. From reading the abstract, 35 articles were selected for full text reading. Of these 35 articles, only seven were selected by inclusion and exclusion criteria. Additional four articles not retrieved through the databases were also selected, but were found in the references of included articles and selected due to their relevance to the study. (Figure 1) Thus, 11 articles were selected to this systematic review. (Tables 1 and 2) Of the 11 articles, seven are case reports.^16^ ^,^ ^19^ ^-^ ^24^ (Table 1) and four articles are retrospective, cohort or prospective studies.^13^ ^,^ ^17^ ^,^ ^18^ ^,^ ^25^ (Table 2)

**Table 1:** Case reports of contamination of the biopsy tract of the musculoskeletal system according to the literature.

Author/year Reference	Nº of cases	Age in years	Gender	Tumor location	Type of tumor	Biopsy technique	Criteria for definition of contamination	CT	∆T	Follow up
Citron *et al*.,^21^ 1984	01	53	M	Lung	Small cell lung carcinoma	Percutaneous^a^	Histology of subcutaneous lesion in biopsy site	Yes^b^	14 months	Disseminated disease
Ginaldi e Williams,^22^ 1985	01	74	M	Lymphatic system	non-Hodgkin Lymphoma	Percutaneous^a^	Histology of lesion in biopsy site	No	11 months	Disseminated disease
Davies, *et al*.,^16^ 1993	01	18	M	Femur distal	Osteosarcoma	Percutaneous	Histology of nodular lesion in biopsy site	Yes	18 months	NI
Schwartz e Spengler,^19 ^1997	03	49	F	Pelvis	Fibro sarcoma	Percutaneous	Tumor histology in the biopsy tract region	No	37 months	NI
		44	F	L4	Pleomorphic skeletal sarcoma	Percutaneous	Histology de satellite tumors along the biopsy tract	Yes^c^	15 months	NI
		56	M	L2	Chordoma	Percutaneous	Histology of relapse tumor on biopsy tract	Yes^d^	21 months	NI
Iemsawatdikul *et al*.,^23^ 2005	01	7	M	Multifocal	Osteosarcoma	Open	Histology of recurring tumor along the biopsy tract	No	NI	Disseminated disease
Fowler, *et al*.,^24^ 2008	02	48	M	Lymphatic system	Follicular Lymphoma	NI^a^	Edema and pain in biopsy site. Biopsy revealed follicular lymphoma	No	10 days	Death
		57	M	Lymphatic system	B cell Lymphoma	NI^a^	Histology of lesion in biopsy site	NI^e^	6 months	NI
Zoccali *et al*.,^20^ 2009	01	47	M	L4	Chondrosarcoma	Percutaneous	Infiltration in the tract detected by NMR	No	1 month	Disseminated disease

CT : Chemotherapy

?t : time interval between biopsy and tract contamination diagnosis

NI : Not informed

M : Male

F : Female

NMR : Nuclear magnetic resonance

L2 : second lumbar vertebra

L4 : fourth lumbar vertebra

CT : computed tomography

a Bone biopsy for staging performed

b Underwent CT for treatment of small cell lung carcinoma

c Underwent radiotherapy and CT for misdiagnosed metastatic carcinoma

d Underwent radiotherapy and CT for misdiagnosed adenocarcinoma

e Underwent CT for treatment of lymphoma. Patient had two biopsies, one before and one after CT, not being clear which one caused tract contamination.

**Table 2:** Cohort studies regarding contamination of the biopsy tract of the musculoskeletal system according to the literature.

Author/year Reference	Nº of cases	Type of tumor	Biopsy technique	Ct	Total contamination in sample	Contamination according to biopsy technique	Contamination according to ct	Criteria to define contamination
Mohana *et al.*, 2007^17^	26	Osteosarcoma	Open 6	Yes^ a^ 24	5 / 26 (19,2%)	Open 2/6 (33.3%)	Yes 3/24 (12.5%)	Histological study of biopsy tract routinely removed during tumor resection
			Percutaneous 20	No 2		Percutaneous 3/20 (15%)	No 2/2 (100%)	
Ribeiro *et al.*, 2009^13^	25	Bone and soft part tumors	Open 7	NI	8 / 25 (32%)	Open 4/7 (57.1%)	-	Histological study of biopsy tract routinely removed during tumor resection
			Percutaneous 18^b^			Percutaneous 4/18 (22.2%)		
Kaffenberg, Wakely Jr and Mayerson, 2010 ^18^	20	Bone and soft part tumors	Open 0	Data does not allow	0	Open -	-	No local remission in non-removed biopsy tract
			Percutaneous 20	analysis^c^		Closed 0		
Saghieh *et al.*, 2010^25^	10	Osteosarcoma and Ewing´s Tumor	Open 0	Yes 10	0	Open -	Yes 0	No local remission in non-removed biopsy tract
			Percutaneous 10	No 0		Closed 0	No -	

CT : Neoadjuvant chemotherapy

NI : Not informed

a Of five cases with contamination, two did not receive neoadjuvant CT due to large tumor extension; 3 were given chemotherapy, but showed poor response. There was no contamination in any case with good response to CT

b All bone tumors underwent percutaneous biopsy and all soft tissue tumors open biopsy by mini-incisions

c authors did not provide clear information about CT, just claim that 16 (80%) of 20 patients received adjuvant and / or neoadjuvant CT

For a better presentation of the results, the articles were divided into two tables. In Table 1 the variables presented are: author, year of publication, number of cases, age, gender, tumor site, type of tumor, biopsy technique, definition of contamination criteria, the time interval between biopsy and contamination diagnosis, and follow up. In Table 2 are presented the variables author, year of publication, number of cases in the sample, type of tumor, biopsy technique, chemotherapy applied, total sample contamination, contamination according to the biopsy technique, contamination according to chemotherapy and definition of contamination criteria.

## DISCUSSION

The first thing to note is the low number of studies in the literature studying the contamination of the biopsy tract by tumor cells in the musculoskeletal system. The heterogeneity of articles does not allow the application of statistical analysis (meta-analysis). In an attempt to trace the profile of patients with contamination of the biopsy tract, it is observed that the case reports addressed 10 cases of contamination of the biopsy tract in the musculoskeletal system. For these cases, the age ranged from 7^23^ to 74 years old.^22^ Eight male patients^16^ ^,^ ^19^ ^-^ ^24^ and two female patients^19^ have been reported. In cohort studies, it is observed that it is not possible to explore the epidemiological characteristics regarding to age and gender, since the authors report these data only for the overall group, not being possible to distinguish between patients who did show and those who showed no contamination of the biopsy tract. These observations reinforce what is perceived in the orthopedic oncology clinical practice, as the literature does not support the possibility of the variables gender or age to influence the occurrence of contamination in the biopsy tract.

The perception that the biopsy tract may be contaminated seems to have been reinforced among the orthopedic community with the work of Cannon and Dyson^15^, who reported a statistically significant lower occurrence of local tumor recurrence where the biopsy tract performed by open technique technical was resected, compared with cases in which it was not resected. It is observed that in none of the 10 cases reported in the selected articles the biopsy tract had been resected. All cases evolved to local relapses.^16^ ^,^ ^19^ ^-^ ^24^ In articles on cohort studies, the work of Kaffenberg *et al*.^18^ and Saghieh *et al*.^25^ the biopsy tract was not removed in any of the patients studied, and there was no local tumor recurrence. In the works of Mohana *et al*.^17^ and Ribeiro *et al*.^13^ all biopsy tracts were removed, and presence or absence of local recurrence was not reported. However, in the work of Mohana *et al*.^17^ five of 26 patients (19.2%) were contaminated in the biopsy tract. On the other hand, on the work by Ribeiro *et al*.^13^ contamination occurred in 25 patients (32%). It is observed by analyzing the literature, that the possibility of local recurrence on unremoved biopsy tract is quite real, the practice of not resecting the biopsy tract appearing not at all safe, despite otherwise shown by Kaffenberg *et al*.^18^ and Saghieh *et al*.^25^

Some authors believe that the percutaneous biopsy technique, by involving less manipulation of tumor tissue, implies in a lower occurrence of contamination in the tract.^4^ ^,^ ^7^ ^,^ ^9^ When analyzing the studies surveyed for this systematic review, it is observed that of 10 reported cases, percutaneous biopsy was performed in seven,^16^ ^,^ ^19^ ^-^ ^22^ open in one case^23^ and in two others the biopsy technique was not informed.^24^ Regarding cohort studies, the work of Mohana *et al*.^17^ reported the occurrence of two cases of contamination in six open biopsies (33.3%) and three contaminations in 20 cases of percutaneous biopsy (15%). No reference was made to the criteria for choosing the biopsy technique, as it was not informed whether the two groups were homogenous. Although many authors believe that the percutaneous biopsy technique has a lower risk of contamination of its path when compared to the open technique, no statistical method was used to test this hypothesis. In the study by Ribeiro *et al*.^13^ four contaminations occurred in seven open biopsies (57.1%) and four in 18 percutaneous (22.2%). The authors also did not perform statistical tests to assess the significance of these differences. It is emphasized that in this work bone tumors and soft tissue tumors were studied, and all bone tumors underwent percutaneous biopsy and all soft tissue tumors underwent open biopsy through mini incisions. Thus, comparing the incidence of contamination between open and percutaneous techniques in this study, it should be noted that the biopsy technique of choice was different for the different types of tumor, making two very heterogeneous groups. In the study of Kaffenberg *et al*.^18^ and in Saghieh *et al.* ^25^ all biopsies were performed by percutaneous technique. In these two studies there has been no contamination in the biopsy tract. Although there is a perception that with the percutaneous technique the chance of contamination is lower, the heterogeneity between studies and the possibility of methodological flaws prevent an accurate conclusion. The main aspect shown in the literature is that tumor contamination in biopsy tract is real even in biopsies performed by percutaneous techniques, reinforcing the need for removal of the path during tumor resection.

Another issue raised in the literature is the influence of tumor type on the occurrence of tumor contamination in the biopsy tract.^13^ ^,^ ^26^ In the ten reported cases , there is a very wide variety in the types of tumors: two cases of osteosarcoma,^13^ ^,^ ^26^ one case of chondrosarcoma,^20^ one case of fibrosarcoma,^19^ one case of pleomorphic sarcoma,^19^ one case of chordoma,^19^ three cases of linfoma^22^ ^,^ ^24^and a case of small cell lung carcinoma ^21^. In these last four types, a bone biopsy for staging the primary tumor was performed. Regarding the cohort articles, Mohana *et al*.,^17^studied osteosarcoma cases and found five contaminations (19.2%) in 26 cases. Moreover, the study of Saghieh *et al.,* in which 25 cases of osteosarcoma and Ewing's sarcoma were analyzed, no contamination occurred. In the work of Kaffenberg *et al*.,^18^ who analyzed various soft tissue and bone tumors reported no contamination. Ribeiro *et al*.,^13^ who also studied bone and soft tissue tumors, found four contaminations (57.1%) in seven soft tissue tumors; and four contaminations (22.2%) in 18 bone tumors. The latter authors suggest that the greater cellularity and smaller amount of matrix, characteristics of soft tissue sarcomas, are related to greater cell spreading compared with bone tumors. It is noteworthy, however, that no statistical test was performed to evaluate the significance of this difference. From the foregoing, it is clear the uncertainty about the influence of the type of tumor on the occurrence of tumor contamination in the biopsy tract in the musculoskeletal system. The great heterogeneity among the studies does not allow a more detailed comparison.

Over the last decades, the treatment of tumors of the musculoskeletal system has been greatly influenced by adjuvant methods. Chemotherapy has been shown to be an effective method in the treatment of some bone tumors, particularly osteosarcoma and Ewing's sarcoma, accounting for a historic change in the prognosis of these tumors, which became more favorable after the introduction of this therapeutic modality.^14^ ^,^ ^27^ ^,^ ^28^ The neoadjuvant chemotherapy administered before surgical resection of the tumor aims to induce tumor regression, allowing a surgical treatment with a lower functional impairment,^27^ ^,^ ^29^ and reduce tumor spread at surgery. Some authors believe that chemotherapy has a protective effect in the control of tumor infiltration in the biopsy site.^17^ ^,^ ^19^ On this issue, the first aspect to be considered is the time that chemotherapy would be administered to have a protective effect. The second issue is that not all tumor types benefit from this therapy. Thus, the study of this protective effect would be unique for tumors amenable to chemotherapy. Furthermore, the sensitivity to chemotherapy is a complex issue with wide variation in the response to each individual patient and for each chemotherapy approach.^26^ ^-^ ^28^ ^,^ ^30^Another issue is that at different times studies used different chemotherapy protocols, also effectively different, making difficult the analysis and comparison between studies. 

By observing this effect of chemotherapy by evaluation of the work selected in this systematic review, we find it extremely difficult to extract the information from the articles. In the seven case reports, in general, the authors did not provide clear information on the administration of chemotherapy. In the 10 cases reported, chemotherapy was not administered in the period between biopsy and the contamination diagnosis in five patients.^19^ ^,^ ^20^ ^,^ ^22^ ^-^ ^24^ In two cases treatment chemotherapy for the primary tumor was done, being one case of osteossarcoma^16^ and one case of small cell lung carcinoma.^21^ In two other cases, chemotherapy was administered in order to treat a tumor which was misdiagnosed.^19^ Thus, given the imprecise efficacy of the chemotherapy protocol employed in these two cases it is impossible to conclude on the possible role of chemotherapy in protecting - or not the tumor contamination . Finally, it is not possible to analyze the role of chemotherapy in one case reported by Fowler *et al.* ^24^ because the patient underwent two biopsies, one before and one after chemotherapy treatment, being not clear in which of them tract contamination occurred. Thus, effectively, only two of the 10 cases reported could likely benefit from chemotherapy protective effect.^16^ In cohort studies, the paper from Ribeiro *et al*.^13^ does not report on the administration of chemotherapy or not, and data from Kaffenberg *et al*.^18^ does not allow any analysis, since the authors state that only 16 (80%) of 20 patients received adjuvant and/or neoadjuvant chemotherapy, without further details . Mohana *et al.* ^17^ observed that the occurrence of tumor contamination in patients receiving neoadjuvant chemotherapy was 12.5% (three of 24 cases). In this study, the two only cases which did not receive neoadjuvant chemotherapy, due to their large tumors, presented with contamination on the biopsy tract. It is noteworthy, however, that the three patients who received neoadjuvant chemotherapy and presented contamination showed a poor response to chemotherapy. In the study of Saghieh *et al*.,^25^ in which neoadjuvant chemotherapy was given to all patients, there was no contamination in the biopsy tract. The studies reveal that although limitations may hinder the assessment of the protective effect of chemotherapy against tumor contamination, the observations of the outcomes of Mohana *et al*.^17^ and Saghieh *et al*.^25^ seem to reinforce the idea that this therapy modality exerts some protective effect against the occurrence of this complication, although other not controlled variables in these studies may jeopardize this conclusion. 

Regarding the prognosis, of 10 cases reported five did not inform follow up.^16^ ^,^ ^19^ ^,^ ^24^ One patient died^24^ and four evolved with spreading of the disease.^20^ ^-^ ^23^ Of the cohort studies, the two studies in which occurred contamination do not mention follow up.^13^ ^,^ ^17^ Although cohort papers do not reinforce this hypothesis, not because they oppose to it, but they do not provide the information, the cases reported in the literature show a strong tendency to the belief that contamination the biopsy tract implies an unfavorable prognosis.

Regarding the criteria for defining contamination, it is observed that most authors used histopathology methods.^13^ ^,^ ^16^ ^,^ ^17^ ^,^ ^19^ ^,^ ^21^ ^-^ ^24^ Well recalled by Ribeiro *et al*.,^13^ when studying biopsy tracts by histopathology methods, a major issue is whether it is possible to pinpoint the location where the biopsy instrument has previously passed through. For this, the authors have suggested the use of local histology alterations, secondary to the aggression promoted by biopsy to the tissue as a marker of the site of biopsy histology.

One aspect that deserves to be recalled is that none of the studies analyzed points out tumor staging as an important factor for contamination in the biopsy tract. Moreover, the range of variables that can interfere with presence or absence of contamination were not or could not be controlled in these studies, making difficult to draw further conclusions.

Several points can be considered regarding the selected works, including the lack of studies with better methodological design. The difficulties seem to be related to the fact that the relative rarity of tumors of the musculoskeletal system and thus, the limitation of samples, the heterogeneity of these tumors and the large number of variables that may interfere with the contamination of biopsy tract by tumor cells. Certainly, these are issues that hinder studies with better methodologies, with standardization of samples and variables control.

## FINAL CONSIDERATIONS

The characteristics of tumor contamination in the biopsy tract in the musculoskeletal system are quite inaccurate according to the literature, although some questions may be raised:

- Age and gender seem to have no influence on the occurrence of this complication;
- In the absence of resection in the biopsy tract, the possibility of local recurrence is quite real;
- It is uncertain the influence of the tumor type on the occurrence of contamination;
- It is not possible to conclude with certainty whether the percutaneous biopsy technique has a lower chance of contamination;
- It is likely that chemotherapy has a protective effect against tumor contamination in the biopsy tract;
- It is expected that patients presenting contamination along biopsy tract evolve with an unfavorable prognosis.
